# Lipid transporter LSR1 positively regulates leaf senescence in Arabidopsis

**DOI:** 10.1080/15592324.2021.2007328

**Published:** 2021-11-22

**Authors:** Guanping Feng, Yihui Zhong, Wenying Zou

**Affiliations:** School of Life Sciences, Jinggangshan University, Ji’an, Jiangxi, PR China

**Keywords:** Leaf senescence, lipid transfer, chloroplast, *LSR1*

## Abstract

Senescence is the final stage in the life history of a leaf, whereby plants relocate nutrients from leaves to other developing organs. Recent efforts have begun to focus on understanding the network-based molecular mechanism that incorporates various environmental signals and leaf age information and involves a complex process with the coordinated actions of multiple pathways. Here, we identified a novel participant, named LSR1 (Leaf Senescence Related 1), that involved in the regulation of leaf senescence. Loss-of-function *lsr1-1* mutant showed delayed leaf senescence whereas the overexpression of *LSR1* accelerated senescence. LSR1 encodes a lipid transfer protein, and the results show that the protein is located in chloroplast and intercellular space. The LSR1 may be involved in the regulation of leaf senescence by transporting lipids in plants.

Leaf senescence is critical for plant fifitness by participating in the orderly disassembly of macromolecules for relocating nutrients from leaves to other organs.^1,2^ Leaf senescence proceeds by integrating various internal and external environmental signals into age information.^1,3^ The external environmental signals, such as pathogen infection, shading, limited nutrients, temperature stresses, and the internal signals, such as reproductive development, reactive oxygen species, various phytohormones, have profound impacts on leaf senescence.^4–7^ The hormones cytokinin and auxin delay senescence, whereas abscisic acid, ethylene, jasmonic acid and salicylic acid accelerate senescence.^8–10^

During senescence, leaf cells are subject to massive structural and biochemical changes in an orderly manner.^1^ Due to the breakdown of chlorophyll accompanied with chloroplast disassembly, the first visible phenotypic change in leaf senescence is the change of the leaf color.^1,3^ The progressive loss of proteins and lipids triggers chloroplast degeneration as well as chlorophyll breakdown. During leaf senescence, enzymes involved in degrading lipid, such as phospholipase D, lytic acyl hydrolase, and lipoxygenase, have a role in the hydrolysis of membrane lipids.^11,12^ For example, plants make use of the lipoxygenase (LOX) pathway to degrade their chloroplasts during leaf senescence. As a member that accumulated in the plastid envelope of the LOX pathway, the 13-LOX catalyzes the dioxygenation of unsaturated membrane fatty acids, resulting in the destruction of the chloroplast.^13^

In higher plants, intracellular lipids trafficking is essential for organelle biogenesis and cell growth. ER and plastid are the two major sites of glycerolipid assembly.^14,15^ Our understanding of how lipids are moved and sorted in plant is very limited, and most of the knowledge about the molecular mechanisms underlying intracellular lipid transport comes from studies in yeast and mammalian.^16^ Trigalactosyl diacylglycerol (TGD) proteins in Arabidopsis has demonstrated their role in polar lipid transfer from ER to chloroplast. The TGD proteins resemble components of a bacterial-type ATP-binding cassette (ABC) transporter. Loss-of-function of the *TGD* genes block lipid transfer from ER to plastids and result in the accumulation of oligogalactolipids.^17,18^

In Arabidiosis, *LSR1* (At1g62500) encodes a lipid transfer protein of 297 amino acids. High-Throughput Single-Cell RNA Sequencing in Arabidiosis root showed that *LSR1* is specifically expressed in the meristematic cortex.^19^ To further expand our knowledge of this lipid transporter, we characterized the T-DNA insertion mutant of *lsr1-1* and generated the transgenic *35S-LSR1* plant. Quantitative RT-PCR analysis showed that *lsr1-1* was knock-out mutant and the expression of *LSR1* was enhanced in *35S-LSR1* transgenic plant. The *lsr1-1* mutant show obvious delayed leaf senescence, whereas overexpression of *LSR1* led to accelerated senescence (Figure 1). Further investigations revealed that the decrease of chlorophyll content and the expression of leaf senescence marker gene *SAG12* were delayed in the mutants and premature in *35S-LSR1* transgenic plants (Figure 2). These results indicate that lipid transporter LSR1 participated in the process of leaf senescence regulation. In Arabidopsis and all other plants, lipids have diverse functions that are essential in cell growth, metabolism, and cell death. Lipid signaling molecules, such as phosphoinositides (PPIns), coordinate numerous aspects of membrane trafficking and cell signaling in eukaryotic cells. LSR1 may be involved in the transport of specific lipid signaling molecules to coordinate the senescence regulation of plant leaves.

**Figure 1.**
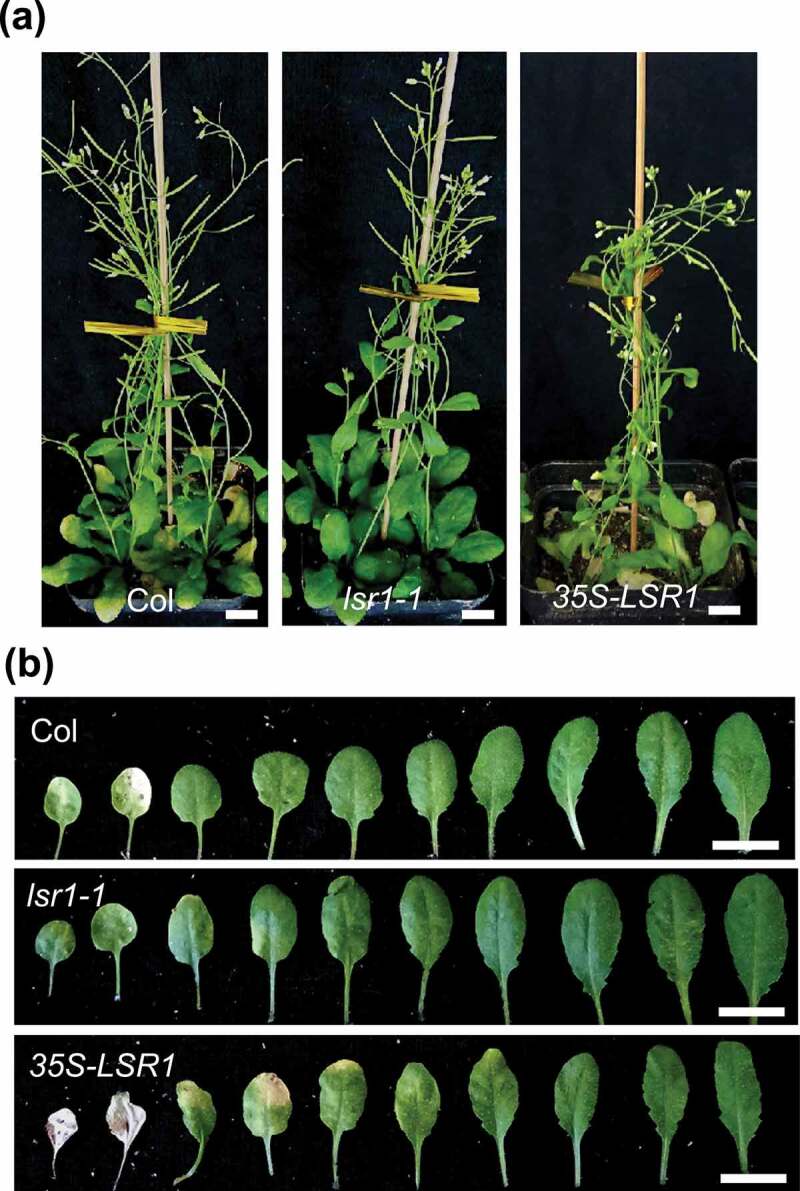
Alteration of the expression of *LSR1* affects leaf senescence. The 45-day-old plants (a) and the fifth leaves (b) of Columbia (Col), *lsr1-1* and *35S-LSR1*. Bar = 1 cm.

**Figure 2.**
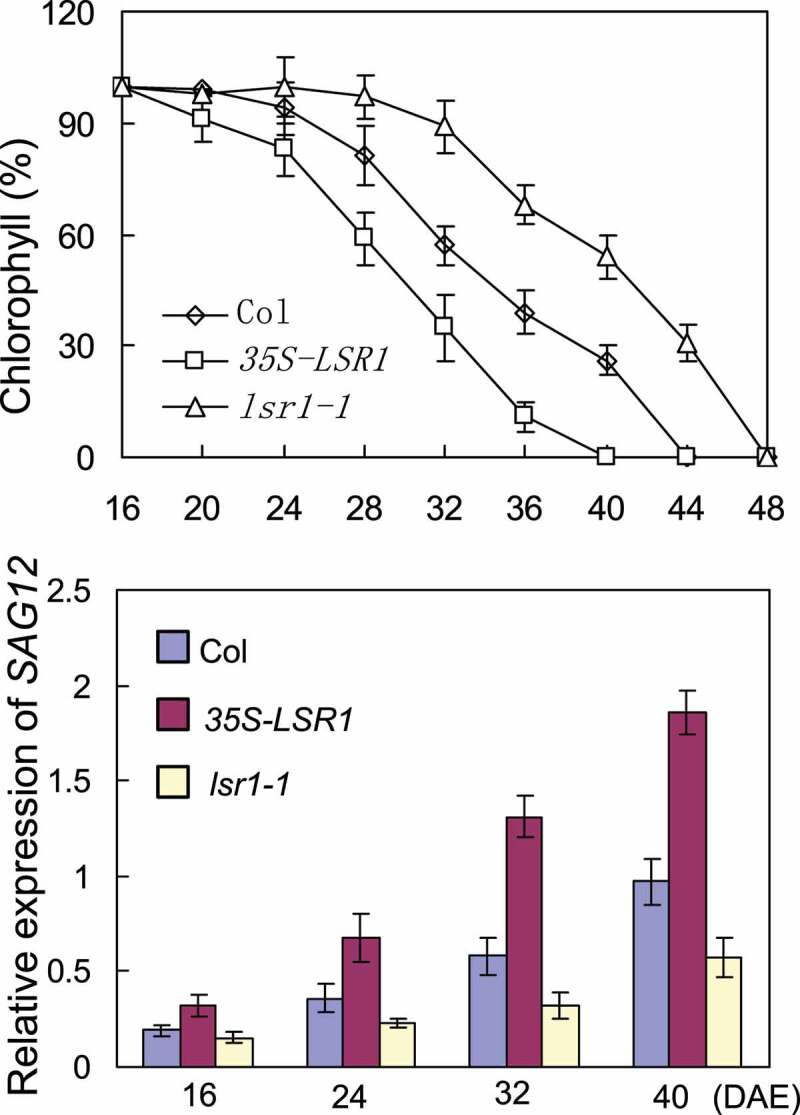
LSR1 positively regulates leaf senescence. (a) Chlorophyll content of the fifth leaves of Columbia (Col), lsr1-1 and 35S-LSR1 at different developmental stages. (b) Expression of SAG12 in Col, lsr1-1 and 35S-LSR1. DAE, days after emergence. Three biological replicates were performed.

To determine the tissue-specific expression of *LSR1*, we generated transgenic plants carrying the *LSR1* promoter::glucuronidase (*proLSR1-GUS*) fusion gene and examined GUS staining. Abundant GUS staining was detected in leaves, especially in vascular tissue (Figure 3). The expression of *LSR1* in leaf is consistent with its role in regulating leaf senescence. To elucidate the biological role of LSR1 as a lipid transporter, we visualized its subcellular localization in transgenic plants carrying *proLSR1::LSR1-GFP*. The GFP florescence signals observed in chloroplast and intercellular space (Figure 4). The chloroplast localization of LSR1 is consistent with the previous reports that lipid transporters are located in plastid. These findings suggest that LSR1 maybe play a role in the transfer of lipid between the chloroplasts. LSR1 may transport specific lipid signaling molecules synthesized in chloroplasts, cross plasma membrane to the extracellular space for long-distance transportation and these lipid signaling molecules are involved in coordinating the regulation of leaf senescence.

**Figure 3.**
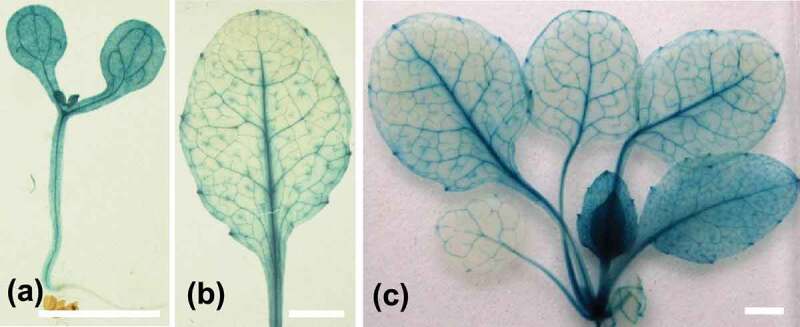
Tissue specific expression of LSR1. GUS activity was assayed in transgenic plants harboring proLSR1-GUS. (a) The 7-day-old seedling; (b) the fifth leaf of 20-day-old plant; (c) the 20-day-old seedling. Bars = 1 cm.

**Figure 4.**
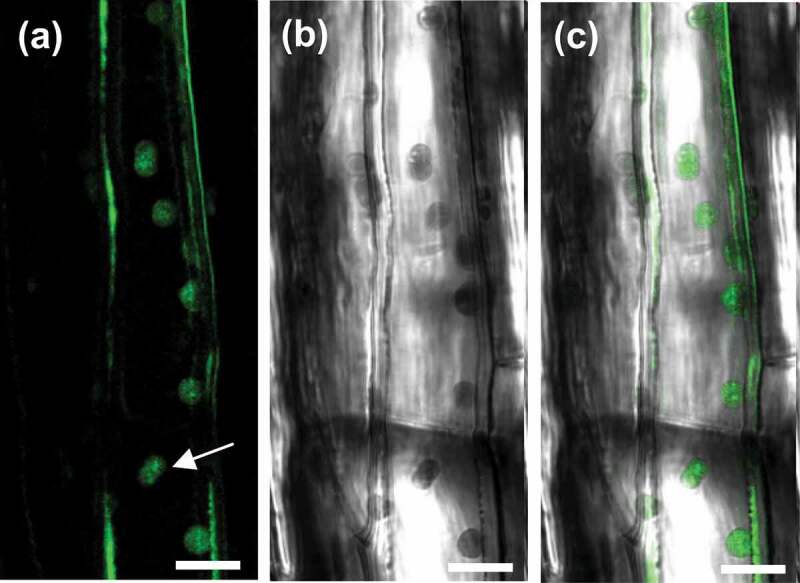
Cellular localization of LSR1 protein. The proLSR1::LSR1-GFP transgenic plants expressing LSR1-GFP fusion protein. The white arrow points to a chloroplast.
